# Future socioeconomic conditions may have a larger impact than climate change on nutrient loads to the Baltic Sea

**DOI:** 10.1007/s13280-019-01243-5

**Published:** 2019-09-21

**Authors:** Alena Bartosova, René Capell, Jørgen E. Olesen, Mohamed Jabloun, Jens Christian Refsgaard, Chantal Donnelly, Kari Hyytiäinen, Sampo Pihlainen, Marianne Zandersen, Berit Arheimer

**Affiliations:** 1 SMHI, 60176 Norrköping, Sweden; 2 Department of Agroecology, Aarhus University, Tjele, Denmark; 3 University of Nottingham, Sutton Bonington, LE12 5RD UK; 4 GEUS, Copenhagen, Denmark; 5 University of Helsinki, P.O. Box 27, 00014 Helsinki, Finland; 6 Department of Environmental Science & iClimate Interdiciplinary Centre for Climate Change, Aarhus University, 4000 Roskilde, Denmark

**Keywords:** Baltic Sea Action Plan, E-HYPE, Hydrological modelling, Nutrient loads, Remedial measures, Water quality, WFD

## Abstract

The Baltic Sea is suffering from eutrophication caused by nutrient discharges from land to sea, and these loads might change in a changing climate. We show that the impact from climate change by mid-century is probably less than the direct impact of changing socioeconomic factors such as land use, agricultural practices, atmospheric deposition, and wastewater emissions. We compare results from dynamic modelling of nutrient loads to the Baltic Sea under projections of climate change and scenarios for shared socioeconomic pathways. Average nutrient loads are projected to increase by 8% and 14% for nitrogen and phosphorus, respectively, in response to climate change scenarios. In contrast, changes in the socioeconomic drivers can lead to a decrease of 13% and 6% or an increase of 11% and 9% in nitrogen and phosphorus loads, respectively, depending on the pathway. This indicates that policy decisions still play a major role in climate adaptation and in managing eutrophication in the Baltic Sea region.

**Electronic supplementary material:**

The online version of this article (10.1007/s13280-019-01243-5) contains supplementary material, which is available to authorized users.

## Introduction

The Baltic Sea Action Plan (BSAP) and the European Union’s (EU) Water Framework Directive (WFD) both require substantial additional reductions of nutrient loads to the marine environment. The Helsinki Commission (HELCOM) estimated that for good environmental status to be achieved, annual reductions of 15,000 tonnes of phosphorus and 118,000 tonnes of nitrogen would be required (HELCOM 2013), as determined from the estimates of loads and environmental objectives for the Baltic Sea for 1997–2003. However, generation and delivery of nutrient loads are strongly affected by the magnitude and seasonality of flows (e.g. Richards and Holloway 1987; Verma et al. 2018), as well as changes in mineralization and denitrification in soil and sediments (e.g. Arheimer et al. 2005; Bouwman et al. 2005).

Changing climate can affect both the flow regime and nutrient sinks and sources in the flow paths. Thus, it is important to understand the magnitude of these proposed reductions within the context of changing climate impact on nutrient loads. Arheimer et al. (2012) analysed climate impacts on the effectiveness of the BSAP by the end of the 21st century, and warned about the potential changing dynamics in stream flow and increased phosphorus loads.

The impact of a changing climate, however, should not be considered in isolation from changing societal drivers. Changes in riverine nutrient loads to the Baltic Sea are determined not only by climate, population, and soil and land use characteristics, but also by technologies adopted in economic sectors, particularly in agriculture and wastewater treatment. Both natural and anthropogenic conditions affect processes such as erosion, deposition, leaching, retention, and transformation of nutrients on the land surface, in the soil subsurface, or in waters.

Only a limited number of studies on nutrient loads have analysed climate impacts combined with comprehensive socioeconomic changes. Most impact studies in the Baltic Sea Drainage Basin (BSDB) evaluated effects of nutrient reduction measures or land use changes (Wulff et al. 2014; Thodsen et al. 2017). Andersson and Arheimer (2003) made a historical reconstruction of nutrient pathways during 100 years of societal changes in a Swedish river basin, while Eriksson Hägg et al. (2014) combined climate projections to the 2100s with scenarios of changes in population and their diet in the BSDB. At a large scale, Seitzinger et al. (2002) evaluated changes in nitrogen loads for three development scenarios (“business as usual” with increased fertilizer use and increased animal proteins in the human diet, “diet” with a lower use of fertilizers and a larger share of plant proteins in the human diet than “business as usual”, and “regional air pollution” with nitrogen depositions reduced due to emission controls and all other inputs the same as in “business as usual”) for North America and Europe, while van Puijenbroek et al. (2015) quantified nutrient emissions from municipal point sources with a country-scale model for two Shared Socioeconomic Pathways (SSPs, O’Neill et al. 2017).

In this study, our objective is to evaluate changes in riverine nutrient loads to the Baltic Sea that can be expected in the 2050s due to changing climate and plausible changes in socioeconomic conditions. For this, we applied scenario modelling and compared results from E-HYPE (Hundecha et al. 2016; Bartosova et al. 2017), a pan-European application of the Hydrological Predictions of the Environment (HYPE) model (Lindström et al. 2010), using different input data representing various climate projections and socioeconomic conditions. We used this relatively high-resolution, distributed, semi-process based hydrological and nutrient model that considers the non-linearity in the response of nutrient dynamics to changing conditions, and simulated both baseline and future nutrient loads to the Baltic Sea by forcing the model with scenarios for future climate and socioeconomic conditions. From this experiment, we could separate the impact of climate change on nutrient load from the impact of socioeconomic factors. The latter affects nutrient load through changes in land use, agricultural practices, atmospheric deposition, and wastewater emissions. The socioeconomic factors can be directly linked to local and regional decision making and are thus highly interesting for nutrient management in the BSDB. 

## Methodology

### Climate and socioeconomics in the 2050s

The new scenario framework developed by the climate change research community over recent years consists of two sets of pathways: Representative Concentration Pathways (RCPs) that describe the extent of climate change and SSPs that depict plausible socioeconomic developments during the 21st century (Riahi et al. 2017). We selected RCP 8.5 (Riahi et al. 2007), one of the more severe climate change pathways, together with SSP1 (Sustainability), SSP2 (Middle of the road), and SSP5 (Fossil-fueled development). The use of SSPs is preferred over extending current trends when evaluating future impacts since future policies, political developments, or other unexpected events may drastically change the direction of current development.

We investigate the importance of these societal developments under one climate pathway (RCP8.5). By mid-century, the differences between RCP4.5—a less extreme pathway—and RCP8.5 are not as pronounced as they become by the end of the century (Hawkins and Sutton 2009). While higher greenhouse gas concentrations (GHC) scenarios are typically not used with SSP1, this combination is plausible by mid-century when taking into account emerging major emission sources (e.g. melting arctic peatland) from positive feedback loops in the natural system (Schuur et al. 2008).

#### Climate forcing data

**Fig. 1:**
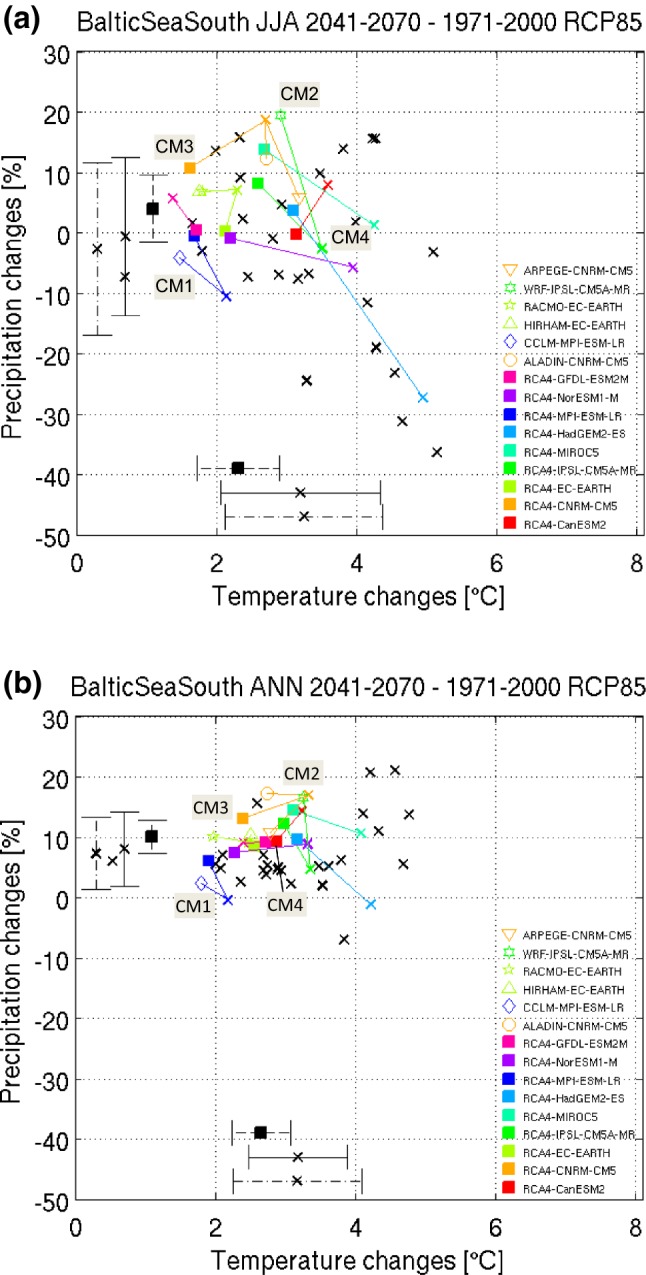
Temperature (°C) and precipitation (%) differences between 2041–2070 and 1971–2000 for **a** summer averages (June–August) and **b** annual averages according to scenario RCP 8.5 for a rectangle encompassing the BSDB south of 60 degrees. Coloured squares show the differences in the RCA4 simulations, open coloured shapes show the differences in the other regionally downscaled simulations. Each regionally downscaled projection is connected by a line to the corresponding GCM, indicated by a coloured cross. Black crosses indicate CMIP5 GCMs not used for downscaling in this study. See Supplementary Materials S1 for details of GCM/RCMs

Full climate ensemble data were collected from Coupled Model Intercomparison Project Phase 5 (CMIP5) projections, downscaled within the Coordinated Regional Downscaling Experiment (CORDEX, www.cordex.org; Jacob et al. 2014). In a pragmatic attempt to maximize the ensemble spread and to minimize the required number of simulations, four climate model (CM) projections with the highest and lowest changes in mean summer temperature and precipitation during a 30-year period around mid-century (2041–2071) were selected (Fig. 1). The changes were determined for a square region encompassing the BSDB south of 60 degrees latitude for each Global Climate Model (GCM) in combination with the Regional Climate Model (RCM) that was used to regionally downscale the GCM. The summer months (June, July and August) were deemed to be the most important for changes in nutrient concentrations due to the importance of plant nutrient uptake. However, the four selected CMs also encapsulate the range of changes seen in the annual averages (Fig. 1b).

Because the GCM/RCM combination CCLM-MPI-ESM-LR (see Supplementary Materials S1 for the lengthy acronyms in this section) represented both the lowest precipitation change and the lowest temperature change, a fourth GCM/RCM combination showing the 2^nd^ lowest temperature change was chosen (RCA4-CNRM-CM5). Also, three models showed very similar changes in temperature but different changes in precipitation. Thus, we chose RCA4-CanESM2 instead of Arpege-CNRM-CM5 to further diversify the GCMs in the ensemble included in the modelling (Table 1).

**Table 1:** The final four chosen climate model (CM) projections for RCP8.5

CM	RCM	GCM	Symbol in Fig. 1
1	CCLM	MPI-ESM-LR	Empty blue rhombus
2	WRF	IPSL-CM5A-MR	Empty dark green star
3	RCA4	CNRM-CM5	Full orange square
4	RCA4	CanESM2	Full red square

See Supplementary Materials S1 for details of individual GCM/RCMs

The selected CMs were then bias-adjusted and downscaled to the required resolution using Distribution Based Scaling (DBS) (Yang et al. 2010). Here, we applied DBS to daily precipitation and temperature using Watch ERA-Interim Forcing Data (WFDEI, Weedon et al. 2011) as a reference dataset. The reference period for the calibration of the bias-adjustment parameters was set to 1991–2010.

#### Shared Socioeconomic Pathways (SSPs)

The changes in the model forcing data were interpreted through three regionally extended SSPs covering socioeconomic drivers that affect nutrient loading to the Baltic Sea (Zandersen et al. 2019). SSPs are used in the global climate research community to explore impacts associated with alternative climate and socioeconomic futures (van Vuuren et al. 2011; O’Neill et al. 2017). SSPs are quantitative and qualitative narratives of plausible socioeconomic futures up to the end of the century. The three SSPs used here are as follows:

SSP1 (Sustainability) describes a world making relatively good progress towards the United Nation’s (UN) Sustainable Development Goals (SDGs) while reducing resource intensity and fossil fuel dependency. The goals of the EU WFD and management plans for reducing nutrient loadings from agriculture would be fully implemented. Consumption trends would change towards less demand for meat. More sophisticated and comprehensive sewage treatment technologies would be adopted. Atmospheric deposition of nitrogen would be reduced following cleaner energy production and use of electric vehicles.

SSP2 (Middle of the road) describes a world where trends typical of recent decades continue with some progress towards achieving SDGs, including reductions in resource and energy intensity and a slow decrease in fossil fuel dependency. Larger farms, intensive farming, and industrialized and more effective agriculture would increase. Management plans (WFD) would be only partly implemented. Sewage treatment technology development and increased urbanization would lead to reduced nutrient loadings. Atmospheric deposition would follow the decrease in NOx emissions as hybrid and electric cars become more widely used.

SSP5 (Fossil-fueled development) is a world that stresses conventional development oriented towards economic growth with a high energy demand mostly met with carbon-based fuels. A global market for agricultural products combined with an increasing global demand for animal products for growing populations would lead to an increase in agriculture and livestock production in the Baltic Sea region. There would be less regulation of agricultural nutrient loadings, but innovations in production technologies may reduce nutrient emissions in relative terms. Increased urbanization and population growth would lead to a higher amount of wastewater, but with higher removal efficiencies due to improved sewage treatment technologies. New technologies to reduce NOx emissions would continue to expand but at a reduced rate compared to SSP1 and SSP2.

SSP1, 2, and 5 were implemented by quantifying changes in nutrient sources (Table 2) based on a spatial interpretation of qualitative narratives (Engardt et al. 2017; Zandersen et al. 2019) and the numerical projections available at the IIASA SSP Database (IIASA 2017). Point source loads reflect changes in the population size, efficiency of sewage treatment, and new investments in infrastructure. Land use and agricultural practices follow changes in population, urbanization, and food production. Note that the SSPs should not be interpreted as “the best case”, “the worst case”, or even as “the most likely case” scenarios as they only represent plausible future developments.

**Table 2:** Main assumptions of socioeconomic impact on nutrient sources and emissions across the Baltic Sea Drainage Basin

Average changes in	SSP1 Sustainable development	SSP2 Middle of the road	SSP5 Fossil-fueled development
Agricultural land use^a^	− 10%	0%	+ 10%
Livestock density	− 50%	0%	+ 50%
Manure nitrogen efficiency	+ 10%	+ 5%	− 10%
Applied effective nitrogen	− 5%	0%	+ 5%
Atmospheric deposition of N	− 40%	− 30%	− 15%
Urban wastewater^b^	− 35%/− 40%	− 20%/− 25%	− 16%/− 23%
Rural wastewater^b^	− 30%/− 30%	− 17%/− 17%	1%/− 23%

^a^Converted to or from forest

^b^The first number refers to changes in N and the second to changes in P where applicable

### Nutrient impact modelling

Daily discharges and nutrient loads for current and future conditions were simulated with E-HYPE for the BSDB. HYPE is an integrated hydrological and nutrient transport model code developed by SMHI (Lindström et al. 2010). E-HYPE, a pan-European model built using the HYPE software, simulates rainfall, runoff, riverine processes, and nutrient processes in hydrologically delineated catchments with a median size of 215 km^2^ for all of Europe. We used E-HYPE v.3.1.4 (Hundecha et al. 2016; Bartosova et al. 2017).

The E-HYPE model v.3.1.4 includes deeper soils with active groundwater (European Hydrogeology map; BGR & UNESCO 2014) and reflects more recent crop distributions (Eurostat 2013) and point source discharges (Urban Wastewater Treatment Directive 2016). While these updates did not significantly affect model performance, we used a stepwise, representative gauged basin (RGB) approach (Strömqvist et al. 2012; Donnelly et al. 2016) to recalibrate selected model parameters that affect nutrient processes. In addition to the typical calibration approach where the outputs are compared to observed concentrations, we also reviewed model performance with respect to three sets of data associated with fundamental hydrological and biogeochemical processes: baseflow fraction in streamflow gauges, nitrogen leaching (Andersen et al. 2016), and the rate of nitrogen reduction in groundwater (Højberg et al. 2017). However, the latter two datasets were based on model analyses and expert judgement with varying spatial resolution and tools and data inputs used to produce the estimates. For example, the rate of nitrogen reduction in groundwater for Sweden was estimated from two different national hydrological models with a median catchment size of 7 km^2^, but for Germany it was estimated as a one constant value for all contributing catchments. Thus, nitrogen leaching and the rate of nitrogen reduction in groundwater were used to guide larger spatial patterns rather than for calibrating individual catchments.

The recalibration focused only on nutrient concentrations; model parameters that affect stream flow remained the same as in the previous E-HYPE version. At the pan-European domain, 46% of the 1015 stream gauges had Nash–Sutcliffe model efficiency (NSE, Nash and Sutcliffe 1970) greater than 0.5 and the relative error (RE) was within 50% at 85% of the gauged locations. The proportion of sites with NSE greater than 0.5 was the same in the BSDB (46% of 368 stream gauges), but a higher proportion of the sites (93%) had a relative error within 50%.

**Fig. 2:**
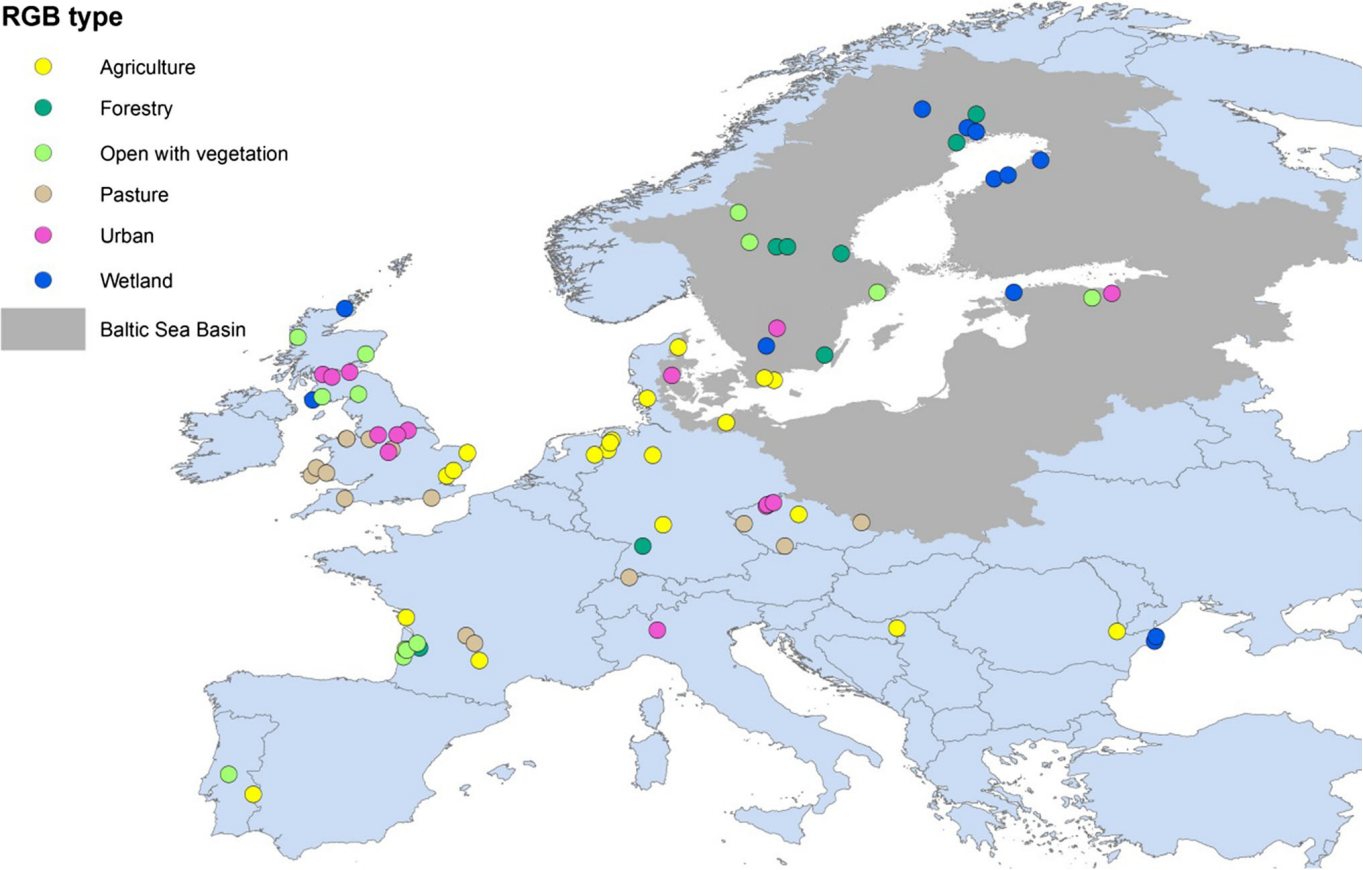
Representative gauged basin (RGB) sites used for recalibration of E-HYPE v. 3.1.4

The E-HYPE model was recalibrated using 89 sites at the full pan-European scale (Fig. 2) because insufficient nutrient observations were available to capture the southern agricultural parts of the BSDB adequately. The calibration period was from January 1, 2001 to December 31, 2010 (10 years) with an initial warm-up period from 1979 to allow the model to achieve stable conditions. The remaining sites were used to validate the model using the same time period.

The relative error (RE) in the recalibrated model varied among the monitoring sites, with 65% and 66% of the sites having RE within 50% for total phosphorus (TP) and total nitrogen (TN) concentrations, respectively, across Europe. The median RE was 8% for TP and − 10% for TN. The recalibration is described fully in Bartosova et al. (2017).

Recalibration resulted in a considerable change in the internal representation of the biogeochemical processes in some areas with a model performance comparable to the original calibration. This is expected to increase the plausibility of the overall model results, especially with respect to nitrogen reduction and retention processes.

### Modelling experiment

Current conditions were simulated with the calibrated model using a 30-year period from 1981 to 2010 (with a 10-year warm-up period from 1971). Future conditions were also simulated using a 30-year period representing the 2050s (from 2036 to 2065 with a 10-year warm-up period from 2027). Time slices were preferred over transient runs as long-term changes in nutrient storage within soils are difficult to validate and can have a large impact on scenario results.

Both current and future conditions were simulated using all four selected CMs. Seven E-HYPE v.3.1.4 model runs were executed with each CM: (1–2) current and 2050s periods with current land use and nutrient sources, (3–5) 2050s period with land use and nutrient sources representing SSP1, 2 and 5, and (6–7) current and 2050s periods with current land use and nutrient sources, but with model parameters prior to recalibration. The last set of model runs was used to test the robustness of the E-HYPE model simulations and the dependency of the results on certain model parameters relevant in nutrient processes.

The model output data were processed in R utilizing the R-package HYPEtools (SMHI 2018). The results from the CMs were then averaged and a relative change from the average of the current period with current land use and nutrient sources (i.e. the first of the seven runs) was calculated. Ensemble-average values for main river basins that discharge directly to the Baltic Sea were summarized to obtain total fluxes to the Baltic Sea.

We also looked closely into how different sectors in SSP2 affect the nutrient loads by simulating four additional sub-scenarios where only one of the following sectors was modified at a time: (1) atmospheric deposition, (2) land use and agricultural practices, (3) point source effluents, and (4) contributions from the rural population (Table 2). SSP2 was selected for this analysis because it is most closely aligned with recent trends in development. In order to enhance insights into the origin of nutrient loads across the BSDB, we conducted a source apportionment analysis for long-term average annual loads from a number of source groups under current and 2050s climate conditions. Modelled loads in HYPE were traced from their origin to user-defined outlet points within the model domain and aggregated as net loads to the Baltic Sea from agriculture, forests, pasture, mixed-use and semi-urban lands, non-forested (semi-)natural lands, rural households, wastewater treatment plants, and industrial effluents.

## Results

### Impact of climate change

**Fig. 3:**
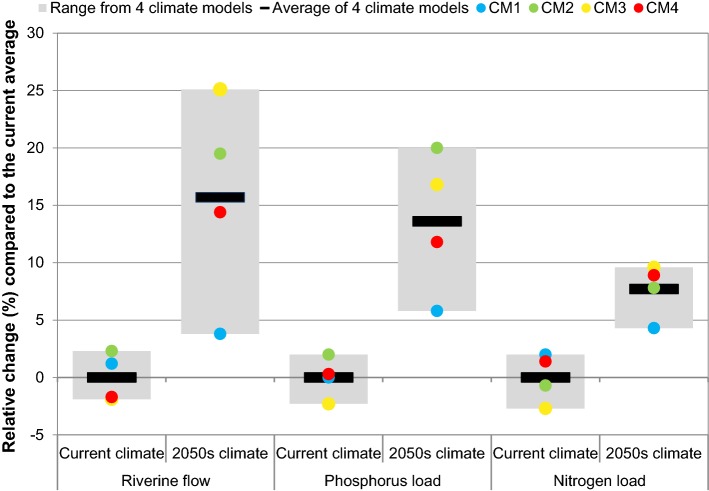
Relative change in flow, phosphorus load, and nitrogen load with respect to the average values during the current climate period

The average simulated TN and TP loads from the BSDB were 540 thousand tons year^−1^ and 29 thousand tons year^−1^, respectively, for the 2001–2010 period. The differences in the current fluxes to the Baltic Sea among the four CMs were minimal, about ± 2% (Table 3 and Fig. 3). Discharge in the 2050s was projected to increase between 4% and 25% with an average increase of 16%. This increase is significantly higher than the variability in modelled flows under current climate due to the CM selection. Seasonal high flows are projected to increase, with peak flows happening earlier for the 2050s (See Supplementary Materials S1).

**Table 3:** Percentage relative change in stream flow and TP and TN loads

	2050s	CM1	CM2	CM3	CM4	Average	Range
Stream flow	v.3.1.4	1.2	2.3	− 1.9	− 1.7	0	4.2
	a	1.2	2.3	− 1.9	− 1.7	0	4.2
TP load	v.3.1.4	5.8	20.0	16.8	11.8	13.6	14.2
	a	3.5	16.2	14.0	9.7	10.8	12.7
TN load	v.3.1.4	4.3	7.8	9.6	8.9	7.7	5.3
	a	5.8	13.7	15.4	10.6	11.4	9.6

^a^Signifies the same E-HYPE model set up with the input files updated as in version 3.1.4 but prior to recalibration of the model parameters (recalibration focused only on nutrient processes, simulated flows are thus the same)

Nutrient loads were projected to increase by 8% and 14% on average for nitrogen and phosphorus, respectively, by 2050s (Fig. 3). This increase is largely associated with the increased total flow. Note, however, that average flow was projected to increase by 16%, which is more than the increase in either of the nutrient loads. This signifies that the average flow-weighted nitrogen and phosphorus concentrations were projected to decrease. Similar increases (11% for both TN and TP loads) were projected with the E-HYPE model runs using the original calibration parameters (Table 3). It is also notable that the variation across the climate models in projections of nutrient loads is smaller than the variation in projected discharges.

### Impact of socioeconomic changes combined with climate change

**Fig. 4:**
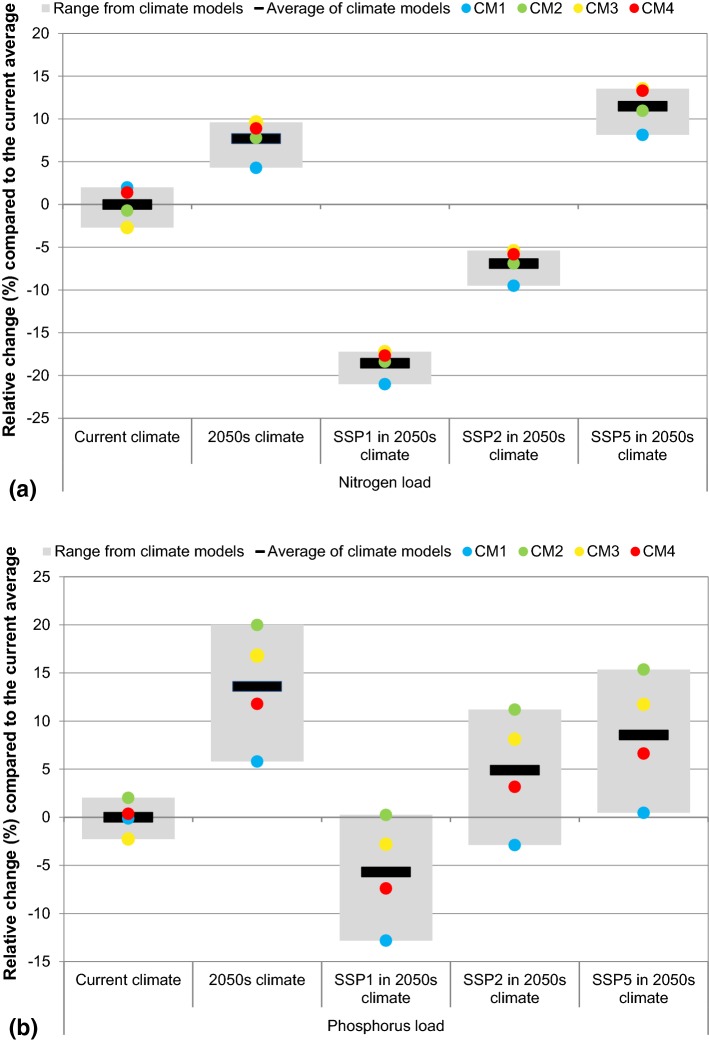
Change in the **a** total nitrogen load and **b** total phosphorus load to Baltic Sea due to changing climate and socioeconomic conditions. Relative change calculated from averages of the four climate models under current climate

The different socioeconomic conditions in the individual SSPs resulted in very different nutrient loads to the Baltic Sea (Fig. 4), especially for nitrogen (Fig. 4a). Under SSP1, the nitrogen and phosphorus loads decreased on average by 19% and 6%, respectively, relative to the current loads despite climate change impacts also being included in the simulations. On the other hand, nitrogen and phosphorus loads under SSP5 increased on average by 11% and 9%, respectively, relative to the current loads.

While all simulations that considered only changing climate resulted in a significant increase in both phosphorus and nitrogen loads (between 6 and 20% and between 4 and 9%, respectively), the combined impact from changing climate and socioeconomics resulted in either significant increases or significant decreases (from − 12 to 15% and from − 21 to 14% for phosphorus and nitrogen loads, respectively) depending on the assumptions concerning socioeconomic development. This significant finding highlights the importance of societal developments.

The impacts from SSPs were not evenly distributed across the BSDB. For example, Northern Sweden showed sustained increases in TN and TP loads for all SSPs considered. As the assumptions progressed from SSP1 to SSP5, more drainage basins switched from showing a decrease in nutrient loads to showing an increase.

The largest variability in projected TN load was observed in the Archipelago Sea where the average impact fluctuated between a 24% reduction under SSP1 and a 62% increase under SSP5, i.e. an overall variability of 86%. The Bothnian Bay was a notable exception in showing an increase in TN loads under SSP1, although this increase was very small (3%). The smallest variability in TN loads was projected for the Gulf of Riga where the impact fluctuated between a 6% reduction under SSP1 and a 12% increase for SSP5.

The river flows for any simulation with both socioeconomic and climate changes were within 1% of those projected for simulations with climate changes only. SSPs were expected to have minimal impact on river flows since, with the exception of land use change, most of the assumptions associated with SSPs affect only nutrients.

### Relative contribution of different sources of nutrients

**Fig. 5:**
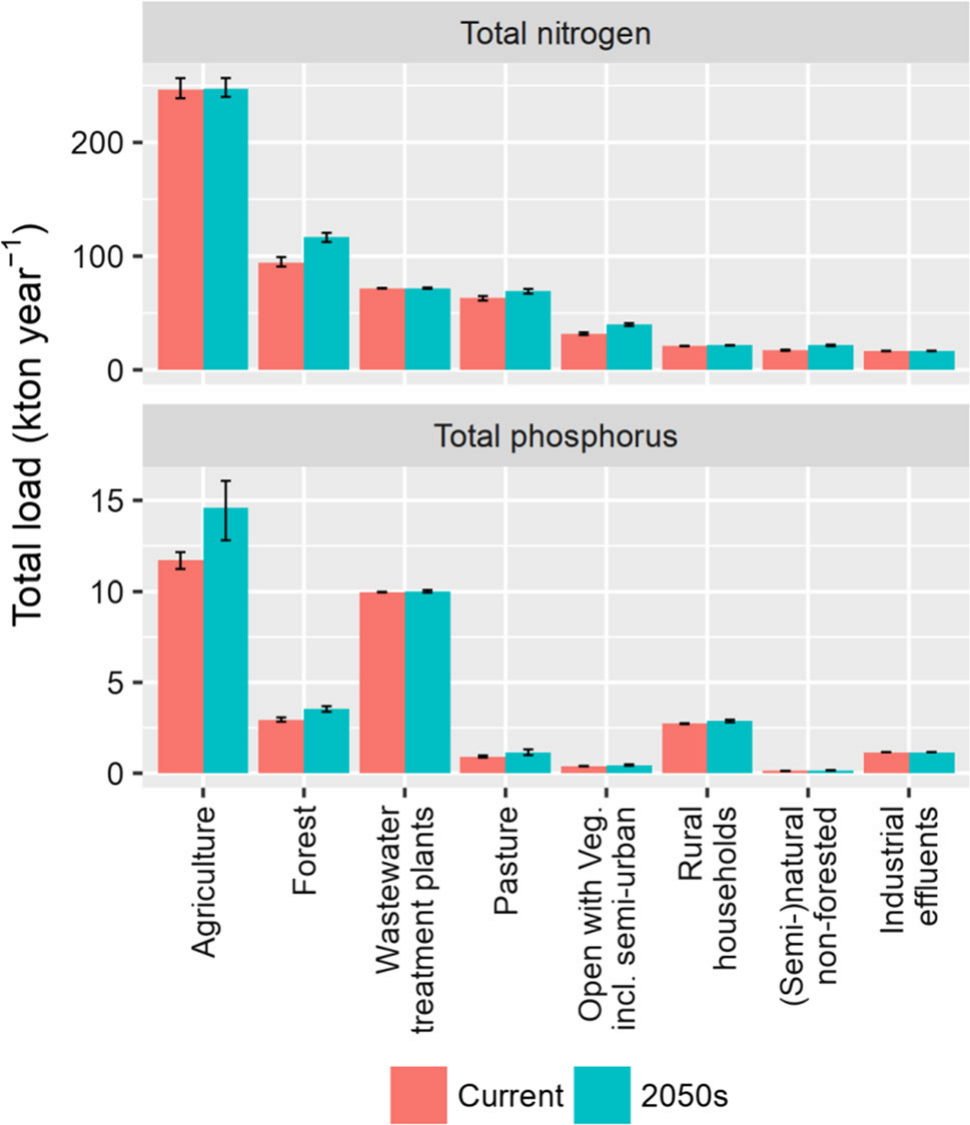
Grouped source contributions to total nitrogen and total phosphorus loads from the Baltic Sea Drainage Basin under current and future conditions. The values represent the average loads under the four selected climate models. Error bars show the range due to variability in the climate models

Nutrient loads to the Baltic Sea originate from multiple sources. The largest current source for both nitrogen and phosphorus loads was found to be diffuse agricultural sources (Fig. 5). Forests, the next largest source of nitrogen load, contributed less than half of the agricultural load. Forests cover a large area of the BSDB and contribute a significant amount of flow with high total nutrient load despite producing typically low nutrient concentrations. For phosphorus, wastewater treatment plants contributed nearly as much as agricultural sources; other sources were much less significant. The variation in simulated loads due to variability in the CMs was most pronounced for agricultural source contributions to phosphorus loads in the 2050s, likely due to changing mobilization through surface erosion and the uncertainty surrounding extreme precipitation event patterns in the CMs.

Overall TN load to the Baltic Sea from agriculture was not projected to change between current climate and the 2050s, although there were regional variations, most notably a decrease in agricultural nitrogen loads from the south-western half of the Baltic Sea region and an increase from other parts. There could be different explanations for this pattern, e.g. increased plant nutrient uptake due to the increasing temperature and longer growing season, higher reduction of nitrogen during subsurface and instream transport due to the combination of low to moderate increases in precipitation with increases in temperature, or simply the differing responses of different agricultural systems and nitrogen processes.

We also analysed the separate impacts of socioeconomic changes that were considered in SSP2 for individual nutrient sources under current climate (see Supplementary Materials S1). These results indicated that the assumed 30% reduction in atmospheric deposition rates reduced current nitrogen loads to the Baltic Sea by about 7%. The changes in agriculture assumed under SSP2, i.e. mostly field management practices, led to more than a 3% reduction in nitrogen load to the Baltic Sea. Urban point sources reduced the total nitrogen load by more than 2% and more than 1%, respectively. For phosphorus, the assumed changes to point sources under SSP 2 dominated, decreasing total phosphorus load to the Baltic Sea by more than 7%. SSP2-assumed changes to rural sources led to about a 1% reduction in phosphorus load, whereas the SSP2 assumptions for changes in agriculture had no, or only negligible, impact on phosphorus load. Atmospheric deposition of phosphorus is not included in E-HYPE v.3.1.4 (Table 2). The sum of the individual changes to nutrient loads projected for SSP2 under current climate and the changes projected under 2050s climate did not match the changes projected for SSP2 under 2050s climate. This was expected, however, due to the interdependence of agriculture and climate, and also due to the non-linearity of formulae in the HYPE model.

## Discussion

### Confidence in results

Estimates of nutrient loads always include uncertainties, as many locations and time-periods in the past remain unmonitored and the future is as yet unknown. Using models is an efficient way to interpolate or extrapolate across space and time, but it should be noted that the results of this model experiment are not predictions or forecasts but only meant for relative comparison of potential impacts of various changes. Nevertheless, comparing our results with those from previous studies and listing some main sources of uncertainty will give an indication of confidence in the results.

The average TP loads simulated with E-HYPE for the BSDB during 2001–2010 are practically the same as the total riverine loads reported by HELCOM (2015) during the same period, with only a 4% difference between the two approaches. For average TN load, the difference of 14% still represents very good agreement considering the difference in approaches as well as the uncertainty in the monitored values.

The modelling chain used in the experiment is a state-of-the-art procedure for climate impact assessments, yet it includes many well-documented sources of uncertainties (e.g. Bosshard et al. 2013; Olsson et al. 2016). GCMs are dynamically downscaled to RCMs with further tailoring via downscaling and bias correction before using the climate data in hydrologic impact models with uncertainties present at each step. For example, Olesen et al. (2019) attributed large differences in the climate impact on TN loads in a Danish catchment (0–8% decrease vs. 23–63% increase) projected by two different hydrologic models at different resolution, despite using the same CMs, to using different reference time series for downscaling the CMs and different evapotranspiration routines, aside from other differences in the models.

The large range of projected impacts in 2050s presented in this study highlights the uncertainty surrounding CMs as the CMs projecting the lowest and the highest changes in precipitation and temperature were selected. It is difficult to say in advance which CM would lead to the most extreme nutrient load projections without actually running the full ensemble. The lowest (6%) and the highest (20%) projected changes in TP loads were found under CM1 and CM2, which feature the lowest and the highest projected change in precipitation and temperature, respectively. However, the highest projected change for TN load (10%) was found under CM3, which had the second highest change in precipitation but a relatively small change in temperature. The narrow range of loads and flows simulated for the current time period documented the adequacy of the DBS method for bias adjustments.

E-HYPE recalibration changed the underlying nutrient processes significantly on a regional scale. However, the direction and the magnitude of the average projected changes are rather similar for the recalibrated and original E-HYPE models: 14% and 11% increase in TP load and 8% and 11% increase in TN load for the recalibrated and original E-HYPE models, respectively. The recalibration did affect the variability of the simulated changes for TN load, however. The range of climate impacts on TN load derived from the recalibrated model (5.3%) was only half of that under the original calibration (9.6%).

The consistency of results under the four different CMs and the two model calibration strategies supports the conclusions of the study with respect to the direction of the projected changes, and provides considerable reassurance and confidence in the study setup. The inherent effects of internal HYPE model processes on the impact assessment could not be evaluated at the BSDB scale without having an ensemble of hydrological models available for comparing the model assumptions.

### Importance of societal impacts

The impact of changing climate on nutrient loads from the BSDB is projected to be rather substantial even by the 2050s. However, our findings suggest that regional changes in societal drivers of nutrient loading (e.g. changes in land use, agricultural practices or wastewater treatment efficiencies) can have effects that are as important as climate change for nutrient loads to the Baltic Sea. This is consistent with Eriksson Hägg et al. (2014) who concluded the lifestyle changes through consumption and population can potentially overshadow the climate effects projected at the end of 21st century with respect to nutrient loads.

The SSPs selected for simulation can be alternatively interpreted as pathways with specific sets of measures that target nutrient sources directly by limiting agricultural activities, atmospheric sources, or wastewater treatment, with the changes in nutrient loads reflecting the efficiency of these measures. SSPs led to clearly deviating trajectories for nutrient loadings. The differences in nutrient loads across studied trajectories are large in comparison to the reduction potential of commonly known nutrient mitigation measures (6% and 19% reduction in phosphorus and nitrogen loads, respectively, under SSP1). For example, stakeholder-selected measures examined by Capell et al. (unpubl. results) resulted in a reduction of between < 1% and 5% for both phosphorus and nitrogen loads from the BSDB (also Hasler et al. 2014; Refsgaard et al. 2019). It is therefore important to continue to address the impacts of agriculture, human waste, and other anthropogenic activities on nutrient loads through management plans and policies.

### Relevance for decision makers

Increased contributions from nutrient sources under 2050s climate highlight the need for adaptation measures to counter the adverse effect of climate change on nutrient loads. Different sources respond differently to climate and societal changes, e.g. reduction in atmospheric deposition and point sources had a comparatively large effect on reducing riverine loads even within SSP2. Agriculture remains a major source of nutrients that may require drastic changes, such as those assumed in SSP1, to achieve the needed reduction.

The recovery efforts outlined, e.g. in the BSAP, need to continue to remedy the eutrophication of the Baltic Sea. The Maximum Allowable Inputs specified in the BSAP are currently exceeded in several Baltic Sea Basins, and the limits will continue to be stretched with changing climate (see Supplementary Materials S1).

The Paris Agreement adopted under the UN Framework Convention on Climate Change sets high expectations for the reduction of future emissions. Its implementation, or a lack of it, can have a direct impact on the Baltic Sea not only through changing climate but also e.g. through changes in atmospheric deposition or more generally through changes in water consumption by various sectors and consequently in water and nutrient cycles.

## Conclusion

We evaluated the change in nutrient loads to the Baltic Sea under changing climate and socioeconomic conditions projected to the 2050s. The results show that:

- The impact of socioeconomic changes can be of the same magnitude, or larger, than the impact of climate change. This provides an indication and a hope that nutrient loads to the Baltic Sea can be reduced, and a direction for policy makers when evaluating the efficiency of mitigation measures and policies.
- The impact of climate change is significant even in 2050s, although we cannot exactly estimate the magnitude of the impact due to the unknown future realization of the climate. This impact needs to be included in policy recommendations for the BSDB.
- Spatial variability and different impacts from different sources need to be considered in management plans because one solution will not fit all areas of the BSDB.

## Electronic supplementary material

Below is the link to the electronic supplementary material.

Supplementary material 1 (PDF 667 kb)
