# Supplementary material for: Future socioeconomic conditions may have a larger impact than climate change on nutrient loads to the Baltic Sea
Source: Ambio. 2019 Sep 21;48(11):1325–36. doi: 10.1007/s13280-019-01243-5 (PMC6814641; doi:10.1007/s13280-019-01243-5)
Supplement: Supplementary file 1 — Supplementary material 1 (PDF 667 kb) [file 13280_2019_1243_MOESM1_ESM.pdf]

Ambio

Electronic Supplementary Material

This supplementary material has not been peer reviewed.

Title: **Future socioeconomic conditions may have a larger impact than climate change on nutrient loads to the Baltic Sea**

Authors: Alena Bartosova, René Capell, Jørgen E. Olesen, Mohamed Jabloun, Jens Christian Refsgaard, Chantal Donnelly, Kari Hyytiäinen, Sampo Pihlainen, Marianne Zandersen, Berit Arheimer

1. Supplementary Materials

1.1. Comparison of nutrient loads with Maximum Allowable Inputs (MAI)

Table 1. Average total nitrogen (TN) and total phosphorus (TP) loads estimated by Baltic HYPE and PLC5.5 (HELCOM 2015) for the period from 2001 to 2010. Maximum Allowable Inputs (MAI) determined for 1997-2003 also include airborne loads (HELCOM 2013). The relative difference between MAI and the sum of Baltic HYPE estimates and atmospheric deposition is shown. Grey highlights show where MAIs are exceeded under current and/or future climate. The total reduction needed by the 2050s can be approximated by adding climate change (CC) impact to the relative difference with MAI. The impact of direct point sources was not quantified, but represents 5% and 7% on average for TN and TP loads, respectively. Light grey highlights show where MAI is threatened if the average impact of direct point sources is considered.

	TP						TN					
	thousand $t.y^{-1}$				% of Baltic HYPE		thousand $t.y^{-1}$				% of Baltic HYPE	
Baltic Sea Basin	Baltic HYPE	PLC5.5	AtmDep.	MAI	Difference with MAI	CC impact	Baltic HYPE	PLC5.5	AtmDep.	MAI	Difference with MAI	CC impact
Baltic Proper	15.8	14.8	1.0	7.4	-56%	15%	282	271	126	325	-20%	3%
Bothnian Bay	1.9	2.3	0.2	2.7	27%	14%	35	49	8	58	35%	25%
Bothnian Sea	2.0	1.8	0.4	2.8	15%	13%	34	50	25	79	35%	21%
Gulf Of Finland	4.2	6.0	0.1	3.6	-18%	13%	64	92	13	102	32%	17%
Gulf Of Riga	2.4	2.4	0.1	2.0	-20%	8%	40	74	10	88	78%	12%
Kattegat	1.0	1.4	0.1	1.7	54%	18%	33	54	20	74	39%	8%
Danish Straits	1.3	1.3	0.1	1.6	12%	13%	55	38	24	66	19%	1%
TOTAL	28.7	29.9	2.1	21.7	-29%	14%	542	628	227	792	6%	8%
Percentage of PLC5.5	96%	100%					86%	100%				

1.2.Changes in Annual Regime

We analysed the annual regimes based on weekly aggregates of E-HYPE model results to illustrate averaged intra-annual dynamics of environmental variables over longer time periods. Averages and percentile ranges (25th to 75th percentile) were calculated from the four CMs, i.e. all four CM runs were concatenated into one long time series and averages and percentile ranges were calculated from this concatenated series. The percentile range thus represents a combination of climate variability within the 30-year period and CM uncertainty. Regimes were analysed for eight rivers representing the Baltic Sea Basins (Figure 1). The largest river from each Baltic Sea Basin was chosen. For the Baltic Proper, the largest Baltic Sea Basin, Helgeå was also included as an additional river draining from south-eastern Sweden. No river basin was included for the Archipelago Sea Basin because of its similarity with neighbouring basins and the lack of a large river.

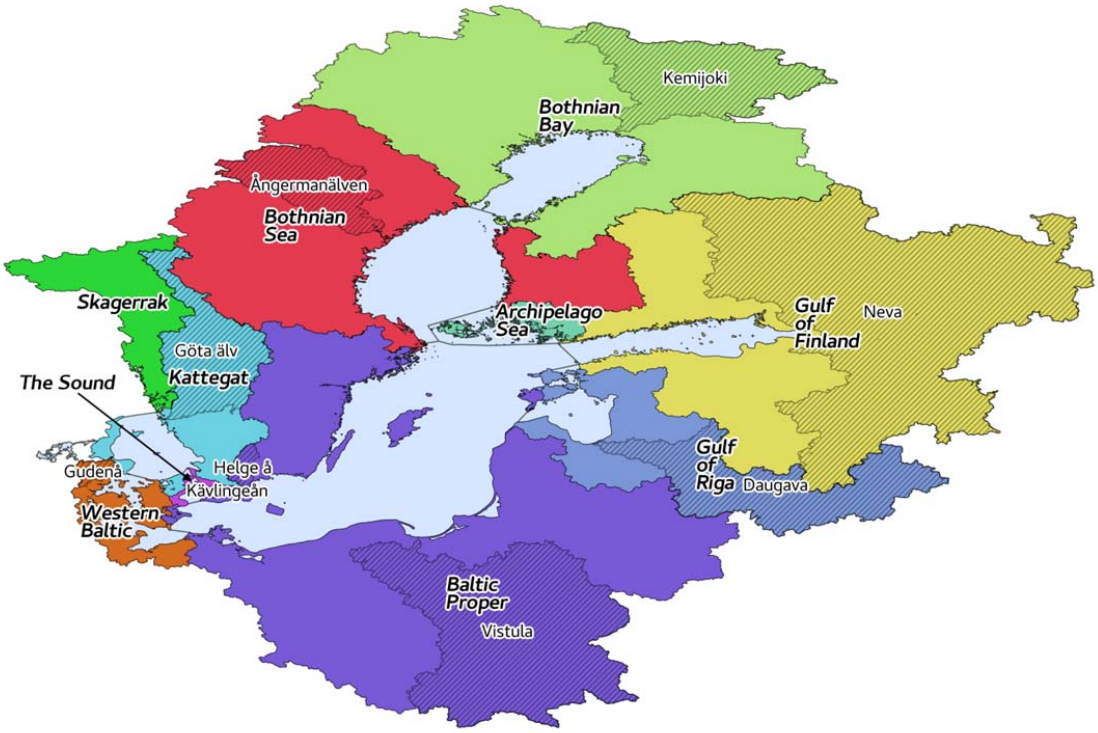

Figure 1. River basins selected for annual regime analysis (hatched overlays), representing individual Baltic Sea Basins (after HELCOM 2018).

A change in streamflow annual distribution was projected for several major rivers in the 2050s (Figure 2). The Helgeå, Vistula (both discharging to Baltic Proper), Kemijoki (discharging to Bothnian Bay), and Daugava (discharging to Gulf of Riga) rivers show pronounced seasonality with high flows during the winter and spring seasons. Seasonal high flows are projected to increase, with peak flows happening earlier for the 2050s. The Neva is highly regulated; seasonality would not be affected under current

regulation, but higher flows would be expected. The feedback linking how changes in natural regime might influence river regulation was not investigated.

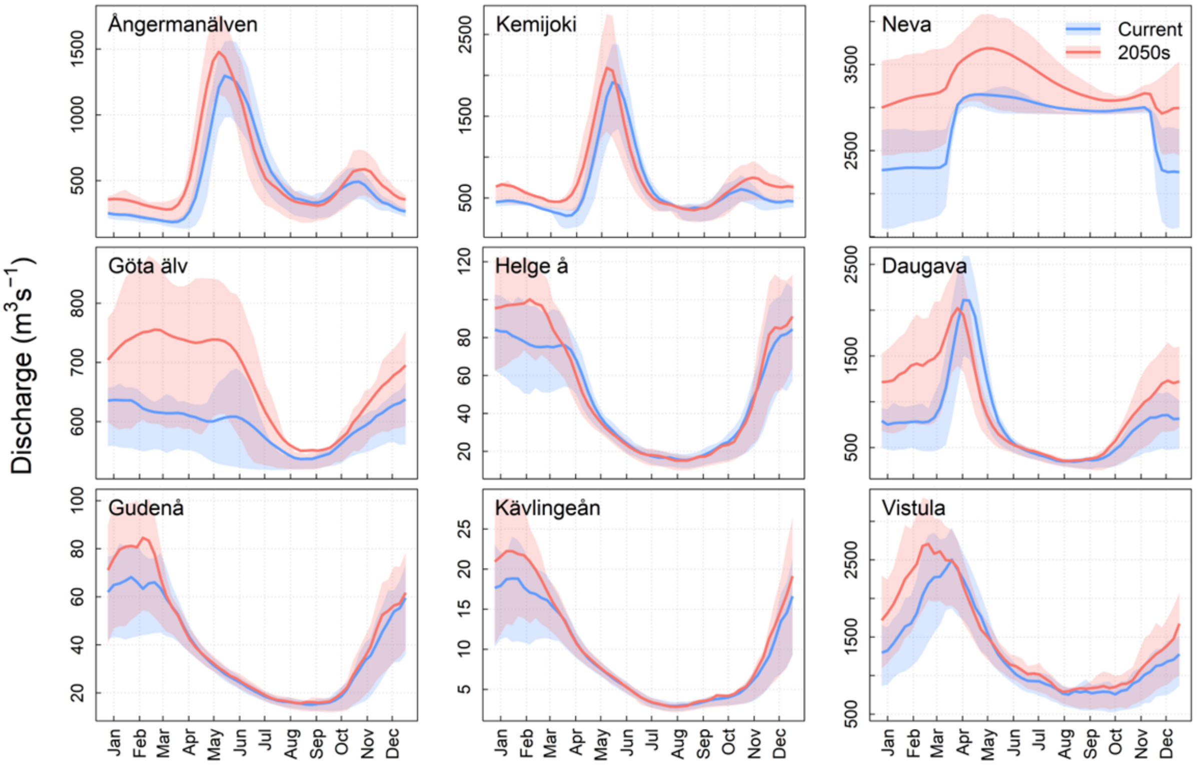

Figure 2. Annual regimes for the Vistula, Helgeå, Neva, Kemijoki, Gudenå, Daugawa, Göta älv, and Kävlingeån rivers under current and 2050s climate conditions for discharge. Note that y-axes on all regime plots are scaled freely, i.e. not fixed between zero and a common maximum value. Each regime plot contains one line together with a transparent ribbon for the current period and another for the future period. Lines show the average values. Ribbons show a 25 % to 75 % percentile range to illustrate inter-annual variation. The size of the ribbon overlap between the current and the future periods is a soft measure of significance of the change signal. It allows the change between the periods to be related to the inter-annual variation within each period.

1.3.Climate Models in CORDEX Used for the Assessment

Global Climate Models (GCMs)

Abbreviation	Full Name
MPI-ESM-LR	Max Planck Institute for Meteorology Earth System Model at low resolution
IPSL-CM5A-MR	Institute Pierre Simon Laplace Earth System Model for the 5th IPCC report at medium resolution
CNRM-CM5	Centre National De Recherches Meteorologiques Climate Model for CMIP5
CanESM2	2nd Generation Canadian Earth System Model by Canadian Centre for Climate Modelling and Analysis
EC-EARTH	European Community Earth-System Model
GFDL-ESM2M	Geophysical Fluid Dynamics Laboratory Earth System Model coupled with Modular Ocean Model
NorESM1-M	Norwegian Climate Centre's Earth System Model
HadGEM2-ES	Hadley's Centre's Global Environment Model 2 - Earth System
MIROC5	Model for Interdisciplinary Research On Climate

Regional Climate Models (RCMs)

Abbreviation	Full Name
CCLM	Consortium for Small-Scale Modelling in Climate Mode (COSMO) Climate Limited-area Modelling
WRF	Weather Research and Forecasting model
RCA4	Rossby Centre regional atmospheric model
Arpege	"action de recherche petite echelle grande echelle" joint model by Météo-France and the European Centre for Medium-range Weather Forecasts (ECMWF)
RACMO	Regional Climate Model by the Royal Netherlands Meteorological Institute and Danish Meteorological Institute
HIRHAM	High Resolution Limited Area Model (HIRLAM) with "Hamburg physics"
ALADIN	"Aire Limitée Adaptation dynamique Développement InterNational" by Météo-France

1.4.Partial Impacts for Socioeconomic Changes in SSP2 under Current Climate

Shared Socioeconomic Pathways (SSPs) were implemented into the E-HYPE model v. 3.1.4 through a set of assumptions (see **Error! Reference source not found.** in the main manuscript). These assumptions affect the following sources of nutrients and inputs to the model:

- Agricultural sources (via field management practices; land use area and livestock density did not change in SSP2),
- atmospheric deposition of nitrogen (atmospheric deposition of phosphorus is not simulated in E-HYPE v. 3.1.4),
- urban point sources (via population change, level of urbanization, and wastewater treatment technology), and
- rural wastewater sources (also via population change, level of urbanization, and wastewater treatment technology).

We analysed how changes in these distinct sources affect TN and TP loads under current climate in order to evaluate how much they individually contribute to the overall change in the loads evaluated in the study. Here we present results for SSP2 (Figure 3 and Figure 4), the pathway that most closely resembles recent trends in socioeconomic development.

Changes in atmospheric deposition are projected to affect the TN load the most under SSP2 (Figure 3), although changes in agriculture are also noteworthy, with the resulting reduction (3.3%) being about half of that for atmospheric deposition (6.5%) despite the model input changes being rather limited. Changes in point sources dominate the change in TP load under SSP2 (Figure 4) and reduce the load by 7.5%. Note that the results in Figure 4 for atmospheric deposition of phosphorus only reflect that there was no change in the model inputs.

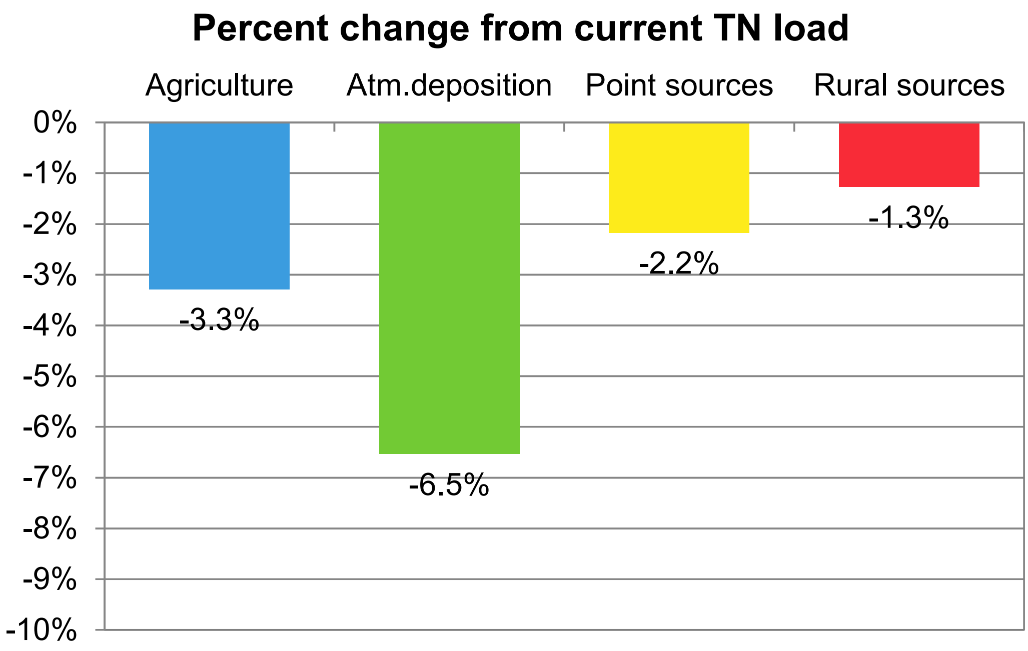

Figure 3. Change in the total nitrogen (TN) load due to changing socioeconomics by nutrient sources. Relative change calculated from averages of the four climate models under current climate.

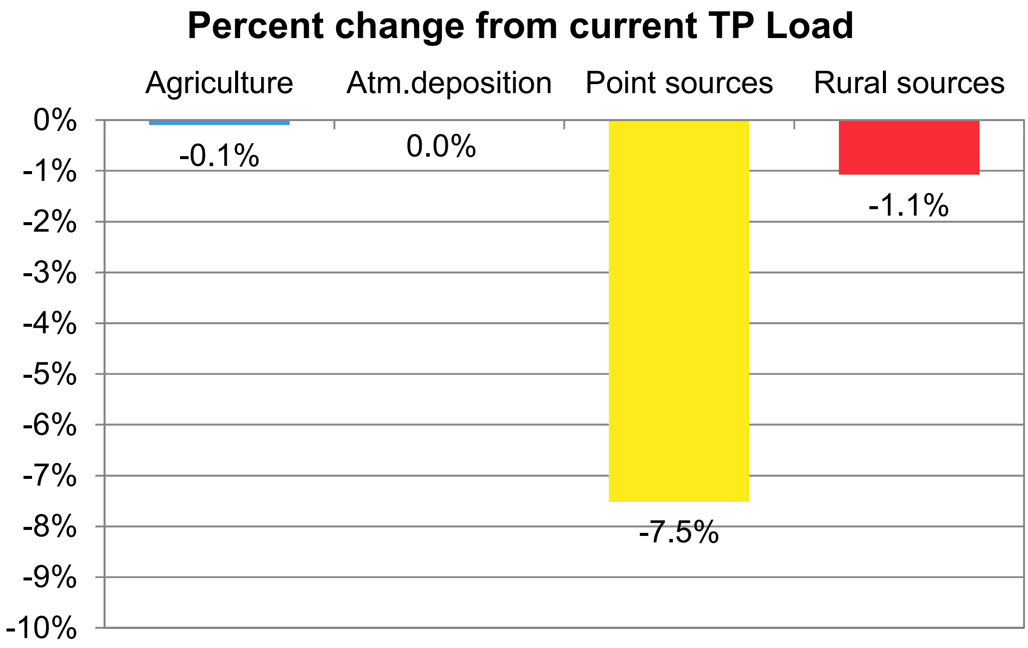

Figure 4. Change in the total phosphorus (TP) load due to changing socioeconomics by nutrient sources. Relative change calculated from averages of the four climate models under current climate.
